# Respiratory Infection in Institutions during Early Stages of Pandemic (H1N1) 2009, Canada

**DOI:** 10.3201/eid1512.091022

**Published:** 2009-12

**Authors:** Alex Marchand-Austin, David J. Farrell, Frances B. Jamieson, Nino Lombardi, Ernesto Lombos, Sunita Narang, Holy Akwar, Donald E. Low, Jonathan B. Gubbay

**Affiliations:** Public Health Agency of Canada, Toronto, Ontario, Canada (A. Marchand-Austin); Ontario Agency for Health Protection and Promotion, Toronto (D.J. Farrell, F.B. Jamieson, N. Lombardi, E. Lombos, S. Narang, H. Akwar, D.E. Low, J.B. Gubbay)

**Keywords:** Influenza, H1N1, disease outbreaks, nursing homes, respiratory tract infections, long-term care facilities, viruses, Canada, expedited, dispatch

## Abstract

Outbreaks of respiratory infection in institutions in Ontario, Canada were studied from April 20 to June 12, 2009, during the early stages of the emergence of influenza A pandemic (H1N1) 2009. Despite widespread presence of influenza in the general population, only 2 of 83 outbreaks evaluated by molecular methods were associated with pandemic (H1N1) 2009.

Respiratory infection outbreaks in institutions housing large numbers of residents create an ideal environment for disease transmission (*1*). Patients in long-term care facilities (LTCFs) for the elderly are more susceptible to respiratory infections and have a higher risk for complications (*2**,**3*).

## The Study

We reviewed respiratory infection outbreaks registered with the Public Health Laboratory (PHL), Ontario Agency for Health Protection and Promotion dating back to October 2007 (Table 1). Molecular detection methods were used for a subset of outbreaks registered during October 1, 2008–April 19, 2009. After emergence of severe respiratory illness clusters in Mexico in early April, intensified tracking of respiratory infection outbreaks in Ontario was undertaken. Consequently, more information was available on outbreaks registered during the spring (April 20 to June 12, 2009); these data comprise the bulk of the study.

**Table 1:** Respiratory outbreak submissions to Ontario, Canada, public health laboratories by geographic location and season*

Region	Influenza season submissions			Spring submissions	
	2007–08	2008–09		2008	2009
Ontario	671	543		117	112†
Greater Toronto area‡	139	101§		34	21

*Influenza season is delineated as October 1–April 19; spring season is delineated as April 20–June 12. †Specimens from 83 of the 112 outbreaks were tested by the RVP assay. ‡Greater Toronto Area includes submissions by Peel, York, and Toronto Public Health Units. §Specimens from 78 of the 101 outbreaks were tested by the Luminex xTAG Respiratory Viral Panel (Luminex Molecular Diagnostics, Toronto, Ontario, Canada).

Respiratory infection outbreaks in LTCFs were defined as any of the following: 2 cases of acute respiratory tract illness, 1 of which was laboratory-confirmed; 3 cases of acute respiratory tract illness within 48 hours in a geographic area (e.g., unit, floor); and >2 units having a case of acute respiratory illness within a 48-hour period. Influenza-like-illness was defined as acute onset of respiratory illness with fever and cough with >1 of the following: sore throat, arthralgia, myalgia, or prostration.

From April 20 through June 12, 2009, a total of 112 respiratory infection outbreaks were registered. Molecular testing was not used in 29 outbreaks (e.g., insufficient/inappropriate sample). Most of the remaining 83 outbreaks submitted for molecular testing originated from LTCFs (91%); hospitals (2%), child care centers (2%), and psychiatric care facilities (1%) comprised the remainder. Facility type was not known for 4% of outbreaks tested. Mean age of persons tested as part of an outbreak investigation was 82 years (SD 13.96 years) and median age was 85 years; 95% were >57 years of age.

Testing on the 589 specimens received from 161 outbreaks registered from October 1, 2008 through June 12, 2009 was performed by real-time reverse transcription–PCR (RT-PCR) for the influenza A virus matrix gene and the Luminex Respiratory Viral Panel (RVP) (Luminex Molecular Diagnostics, Toronto, Ontario, Canada) for other respiratory viruses.

An etiologic agent was identified in 89% of the 161 outbreaks tested by molecular methods. One-hundred-eleven (69%) were caused by 1 etiologic agent. Two and 3 different pathogens were identified in 24 (15%) and 6 (4%) outbreaks, respectively. Four pathogens were identified in 2 (1%) outbreaks. No etiologic agent was identified in 18 (11%) of the outbreaks tested by molecular methods, which includes 1 specimen in which the result was indeterminate for coronavirus OC43. A wide range of causative etiologic agents were detected for outbreaks by the RVP assay (Table 2). Specimens from most patients were positive for enterovirus/rhinovirus (114 patients) followed by metapneumovirus (85), parainfluenza virus type 3 (55), and human influenza virus A (H3) (41). No virus was identified in 186 patients.

**Table 2:** Etiologic agents identified by the Luminex Respiratory Virus Panel* from samples submitted by regional health units during outbreaks, Canada†

**Etiologic agent**	2009 spring outbreaks, Ontario, no. (%)	2009 spring outbreaks GTA,‡ no. (%)	2008–2009 influenza season outbreaks, GTA,‡ no. (%)
**Coronavirus OC43**	1 (1)	0	18 (23)
**Coronavirus NL63**	0	0	6 (8)
**Coronavirus 229E**	4 (5)	0	9 (12)
**Metapneumovirus**	17 (20)	2 (12)	21 (27)
***Respiratory syncytial virus A***	0	0	5 (6)
***Respiratory syncytial virus B***	1 (1)	0	17 (22)
**Influenza A (H3, human)**	11 (13)	6 (35)	4 (5)
**Parainfluenza 1**	1 (1)	0 (0)	1 (1)
**Parainfluenza 3**	22 (27)	7 (41)	3 (4)
**Enterovirus/rhinovirus**	31 (37)	3 (18)	15§ (19)
**Pandemic (H1N1) 2009 virus**	1 (1)	1 (6)	0
**Invalid test¶**	0	0	1 (1)
**None**	6 (7)	0	11 (14)
**Outbreaks tested**	83	17	78

*Luminex Molecular Diagnostics, Toronto, Ontario, Canada. **†GTA, Greater Toronto area. Spring season is delineated as April 20–June 12; influenza season is delineated as October 1–April 19.** **‡Iincludes submissions by Peel, York, and Toronto Public Health Units only.** **§Seven of the 15 outbreaks were confirmed as rhinovirus by the Seeplex RV12 detection kit (Seegene, Inc., Seoul, South Korea).** **¶Reported when the internal control is not detected during a run.**

Co-infections were noted in 22 of the patients tested by the RVP assay. In 1 LTCF outbreak, 2 patients had co-infection of an untypeable influenza A and enterovirus/rhinovirus on testing by RVP. An influenza A real-time RT-PCR result was negative in both patients; 1 patient had a co-infection with respiratory syncytial virus B and enterovirus/rhinovirus. Co-infections with coronavirus subtypes 229E and NL63 were the most common, observed in 10 of the 22 patients (45%) infected with multiple pathogens. Isolates from 1 patient were positive for 3 viruses (coronavirus subtypes 229E and NL63 and enterovirus/rhinovirus).

One of the 2 outbreaks identified as caused by pandemic (H1N1) 2009 originated from a LTCF was not observed until June 3, 2009, six weeks into the evolving pandemic, despite widespread community prevalence. The second pandemic (H1N1) 2009 outbreak, registered on June 11, 2009, originated from a hospital treating patients with influenza-like illness. Seasonal influenza (H1N1 and H3N2) or pandemic (H1N1 2009) was detected in 2,966 (25.5%), and pandemic (H1N1) 2009 in 2,203 (19%) of 11,612 persons tested at PHL for influenza A by real-time RT-PCR during April 20–June 12, 2009. Seasonal influenza A (H3N2) was only identified in 273 specimens (11.0%) of the 2,476 influenza A positive samples subtyped. However, it was the strain responsible for 15 (88%) of the typeable influenza A outbreaks at the same time. Seasonal influenza A (H1N1) was absent from institutional outbreaks and only detected in 41 (2%) of subtyped influenza A–positive samples from the general population.

Persons with laboratory-confirmed pandemic (H1N1) 2009 infection tested at the PHL, Ontario Agency for Health Protection and Promotion, were younger than those tested as part of outbreak investigations. Mean and median ages were 21.5 and 16 years, respectively; only 10% were >46 years of age.

## Conclusions

The number of respiratory infection outbreaks in institutions submitted to PHL may reflect disease impact caused by respiratory viruses during the influenza season. Respiratory viruses during the 2007–08 season may have been more active than those of the 2008–09 season because the number of outbreaks registered with PHL decreased from 1 year to the next. Declaration of pandemic status for the novel (H1N1) virus has not influenced the reporting of respiratory infection outbreaks from institutions in Ontario because submission rates for the corresponding period in 2007–08 and 2008–09 are similar. Variation would not be expected because reporting is required by Ontario law (*4*).

Respiratory viruses detected in outbreaks in institutions reflect those known to be major causes of acute respiratory disease in the community; prevalence varies based on geographic location, season, and detection methods (*5**–**7*). Free access to such institutions by members of the community (staff or visitors), in conjunction with communal close quarters of residents, creates an ideal environment for propagation of viral respiratory outbreaks (*8*).

Current guidelines for isolation during viral respiratory outbreaks are not tailored for the specific virus. As shown in this study, multiplex molecular testing makes it possible to identify the virus causing most LTCF respiratory infection outbreaks. Infection control guidelines for a specific outbreak could be modified based on the incubation period and duration of viral shedding for the identified virus (*9*).

The most commonly identified virus in our study was enterovirus/rhinovirus. Clinicians should be reminded that rhinovirus can cause severe lower respiratory tract infection, including death, as documented in several LTCF outbreaks (*10**,**11*). These data highlight the need for molecular capacity to diagnose rhinovirus infection because detection is otherwise limited to less sensitive viral culture systems.

This review of outbreaks predominantly involving elderly persons in LTCFs highlights the sparing of older persons by pandemic (H1N1) 2009. Possible explanations include cross-protective antibodies from previous exposure to influenza A (H1N1) strains circulating before the antigenic shift of influenza A to subtype H2N2 in 1957 or minimal contact with those most likely to have imported the pandemic strain into Canada (young travelers) (*12*). In addition, older persons may have less contact with the age group (children 10–19 years of age), with most cases being in Ontario. Our findings support Centers for Disease Control and Prevention guidelines for vaccination with monovalent pandemic (H1N1) 2009 virus vaccine. These guidelines have not placed older persons in a high priority group for vaccination because increased rates of hospitalization and severe disease caused by pandemic (H1N1) 2009 have not been observed (*13**,**14*). Investment in multiplex technologies to investigate respiratory outbreaks in LTCFs shortens time for pathogen detection, helps guide infection control and vaccination policies, and can potentially save resources spent on other investigations.
